# HIV-1 Diversity, Transmission Dynamics and Primary Drug Resistance in Angola

**DOI:** 10.1371/journal.pone.0113626

**Published:** 2014-12-05

**Authors:** Inês Bártolo, Suzana Zakovic, Francisco Martin, Claudia Palladino, Patrícia Carvalho, Ricardo Camacho, Sven Thamm, Sofia Clemente, Nuno Taveira

**Affiliations:** 1 Unidade dos Retrovírus e Infecções Associadas, Centro de Patogénese Molecular e Instituto de Investigação do Medicamento (iMed.ULisboa), Faculdade de Farmácia, Universidade de Lisboa, Lisboa, Portugal; 2 Centro de Investigação Interdisciplinar Egas Moniz, Instituto Superior de Ciências da Saúde Egas Moniz, Monte de Caparica, Portugal; 3 Laboratório de Biologia Molecular, Centro Hospitalar Lisboa Ocidental, Hospital Egas Moniz, Lisboa, Portugal; 4 Rega Institute for Medical Research, KU Leuven, Leuven, Belgium; 5 Abbott GmbH & Co. KG, Wiesbaden, Germany; 6 Hospital da Divina Providência, Serviço de Doenças Infecciosas, Luanda, Angola; Institut Pasteur, France

## Abstract

**Objectives:**

To assess HIV-1 diversity, transmission dynamics and prevalence of transmitted drug resistance (TDR) in Angola, five years after ART scale-up.

**Methods:**

Population sequencing of the *pol* gene was performed on 139 plasma samples collected in 2009 from drug-naive HIV-1 infected individuals living in Luanda. HIV-1 subtypes were determined using phylogenetic analysis. Drug resistance mutations were identified using the Calibrated Population Resistance Tool (CPR). Transmission networks were determined using phylogenetic analysis of all Angolan sequences present in the databases. Evolutionary trends were determined by comparison with a similar survey performed in 2001.

**Results:**

47.1% of the viruses were pure subtypes (all except B), 47.1% were recombinants and 5.8% were untypable. The prevalence of subtype A decreased significantly from 2001 to 2009 (40.0% to 10.8%, P = 0.0019) while the prevalence of unique recombinant forms (URFs) increased>2-fold (40.0% to 83.1%, P<0.0001). The most frequent URFs comprised untypable sequences with subtypes H (U/H, n = 7, 10.8%), A (U/A, n = 6, 9.2%) and G (G/U, n = 4, 6.2%). Newly identified U/H recombinants formed a highly supported monophyletic cluster suggesting a local and common origin. TDR mutation K103N was found in one (0.7%) patient (1.6% in 2001). Out of the 364 sequences sampled for transmission network analysis, 130 (35.7%) were part of a transmission network. Forty eight transmission clusters were identified; the majority (56.3%) comprised sequences sampled in 2008–2010 in Luanda which is consistent with a locally fuelled epidemic. Very low genetic distance was found in 27 transmission pairs sampled in the same year, suggesting recent transmission events.

**Conclusions:**

Transmission of drug resistant strains was still negligible in Luanda in 2009, five years after the scale-up of ART. The dominance of small and recent transmission clusters and the emergence of new URFs are consistent with a rising HIV-1 epidemics mainly driven by heterosexual transmission.

## Introduction

Despite the recent decline in the number of people newly infected with HIV, around 35.3 million people were still living with HIV at the end of 2012 [1]. Sub-Saharan Africa remains severely affected by the epidemic accounting for 71% of the people living with HIV in the world and for 69.5% of the new infections [1].

Angola is a South-western African country bordered by Republic of Congo, Democratic Republic of Congo, Zambia and Namibia. According to the UNAIDS report on the global AIDS epidemic 2013 [1] the estimated HIV prevalence and new infections in adults have decresead between 2001 and 2012 in all the bordering countries of Angola. For example, in the Republic of Congo HIV prevalence decreased from 4.7% to 2.8% and the number of new infections decreased from 6,600 to 3,400. In contrast, the estimated number of adults living with HIV in Angola has increased in the same period from 110,000 to 220,000 (1.8% vs 2.3% prevalence) and the estimated number of new infections rose from 16,000 to 23,000 [1]. However a recent HIV seroprevalence survey performed on pregnant women in 36 sentinel sites in 18 provinces of Angola has found that on aggregate HIV prevalence did not vary significantly from 2004 up to 2011 (median 2.8%, range 2.7%–3.2%) although there was considerable variation across provinces [2]. Additional studies are clearly needed to better characterize the dynamics of the HIV epidemic in Angola.

HIV-1 epidemic in Angola is highly complex with all HIV-1 group M subtypes (except B), several circulant recombinant forms (CRFs), unique recombinant forms (URFs) and untypable (U) strains reported [3], [4], [5], [6]. This genetic complexity may pose a significant challenge to laboratory diagnosis and antiretroviral treatment (ART) effectiveness [7], [8], underscoring the importance of implementing regular surveys of HIV-1 diversity and its impact in this country.

Transmitted drug resistance (TDR) is a major public health problem, especially in resource-limited settings as it can determine rapid loss of effectiveness of first-line antiretroviral (ARV) regimens [9], [10]. Drug-naive individuals that acquire a virus with drug resistance mutations (DRMs) begin ART with a higher risk of virologic failure and of developing resistance [9], [11]. The absence of proper patient monitoring may lead to increased emergence and transmission of resistant strains [12]. ART has been available in Angola since 2000 for those infected with HIV who could buy ARV drugs. Since 2004, a national plan has been implemented to provide free ARV drugs to HIV-1 infected individuals using the WHO public health approach to ARV delivery [13]. At the end of 2012 the number of people on ART was 39,704 [1], 48% of the adults in need of treatment based on WHO 2010 guidelines [14]. The frequency of TDR in Angolan patients has risen from 1.6% in 2001 [15] to 16.3% in 2008–2010 [4] suggesting that TDR may be an important public health problem in Angola. However, further work is required to characterize TDR level in Luanda as only a few patients living in this province have been included in previous surveys.

In this study we aimed to better characterize the genetic diversity of HIV-1 and determine the prevalence of TDR in drug-naive patients in Luanda five years after ART scale-up in 2009. Additionally, to better understand the dynamics of the HIV-1 epidemics we performed the first investigation of HIV-1 transmission networks in Angola.

## Materials and Methods

### Study population

One hundred and thirty nine plasma samples were collected during 2009 from drug-naive HIV-1 positive individuals attending the Hospital da Divina Providência (HDP) in Luanda, Angola. This hospital is located in the Kilamba-Quiaxe district serving an estimated population of 990,892 inhabitants, 13.4% of Luanda's population (7,395,977 habitants) [16]. Besides the patients attended at the main building, the hospital works with patients attending four health centers located in different regions of Luanda. The main criteria for patient inclusion in the study were those recommended by the WHO for this type of study [12]: confirmed diagnosis of HIV-1 infection, no pregnancy or first pregnancy (to exclude previous use of ARV for the prevention of mother-to-child transmission during delivery), no clinical diagnosis of AIDS (stage 1 and 2 WHO classification system for HIV infection) and no ART exposure. Epidemiological, clinical, and virological characterization of the patients is given in Table 1.

**Table 1:** Epidemiological, clinical, and virological characteristics of HIV-1 Angolan patients analyzed in this study.

Variables	Samples
Patients [n (%)]	139 (100)
Age [mean (SD), years]	36 (14) (n = 139)
Gender [n (%)]	
Male	50 (36.0)
Female	87 (62.6)
Unknown	2 (1.4)
Transmission route [n (%)]	
Heterosexual	120 (86.3)
Vertical	14 (10.1)
Blood transfusion	2 (1.4)
Unknown	3 (2.2)
CD4 [mean (range), cells/µl]	240.5 (1–1914) (n = 106)
HIV RNA [mean, (SD) log_10_, copies/ml]	5.1 (1.0) (n = 86)
Pure subtype [n (%)]	65 (47.1) (n = 138)
Untypable	8 (5.8) (n = 138)
Recombinants [n (%)]	65 (47.1) (n = 138)

Serological diagnosis of HIV-1 infection was done using the rapid tests Determine HIV-1/2 (Abbott) and Uni-Gold Recombigen (Trinity Biotech). The number of CD4^+^ T cells was determined using the ABACUS 5 Junior Hematology analyzer. Plasma viral load was determined in a subset of patients using the Abbott Real Time HIV-1 assay (Abbott Laboratories).

The study was conducted according to the Declaration of Helsinki and was reviewed and approved by the Board of Directors of Hospital da Divina Providência (Luanda, Angola) and the National Ethics Committee of Angola. Written informed consent was obtained from all participants. The study was verbally explained to the patients before they signed the written consent.

For the transmission network study, to avoid overestimation of relatedness between sequences due to the use of scarce data [17] we extended the study population to all other Angolan patients for which *pol* sequences were available in the Los Alamos HIV Sequence Database [18]. Hence, in addition to our present sequences we used 226 Angolan *pol* sequences collected from the Los Alamos HIV Sequence Database, counting in total 364 sequences. These sequences were derived from samples collected in 1993 and 2001 (n = 86) [6], 2009 (n = 39) [18] and 2008-2010 (n = 101) [4]. Most sequences (n = 64, 28.3%) were obtained from patients attending different medical facilities in and near Luanda (including Hospital Sanatório de Luanda, Laboratório da Força Aérea Nacional Angolana, Hospital Militar Principal, Clínica Sagrada Esperança, Centro Nacional de Sangue and São Lucas Medical Center in the village Kifangondo). Remaining sequences were obtained from patients attending Hospital services in Cabinda (n = 20, 8.8%), Namibe (n = 4, 1.8%), Benguela (n = 4, 1.8%), Zaire (n = 3, 1.3%), Cuanza Norte, Bengo and Huila (n = 1, 0.4%, each), and from patients living in Central (n = 7, 3.1%), North (n = 3, 1.3%) and South (n = 2, 0.9%) of Angola. Origin of 116 (51.3%) patients was not available. Fourteen patients were on ART. Because HIV transmission networks are mainly confined to a country [19] no sequences outside Angola were included in the present study.

### Viral RNA extraction, PCR amplification and sequencing

Viral RNA was extracted from 140 µl plasma using QIAmp Viral RNA Mini Kit (Qiagen). RT-PCR was performed with Titan One Tube RT-PCR System (Roche). Nested PCR was done using an in-house method described elsewhere [15], [20]. Thermal cycling conditions for PCR and primers sequence and position were previously described [15], [20]. DNA sequences were obtained with Big Dye Terminator Cycle Sequencing Kit (Applied Biosystems) and an automated sequencer (3100-Avant Genetic Analyzer, Applied Biosystems).

### Phylogenetic and recombination analysis

Sequences were aligned with reference strains collected from the Los Alamos HIV Sequence Database [18] using ClustalX [21]. Maximum-likelihood (ML) phylogenetic analyses [22] were performed using the best-fit model of molecular evolution estimated by Modeltest v3.7 under the Akaike information criterion [23]. ML trees were inferred, with program PhyML using Seaview software [24]. To find the ML tree, an iterative heuristic method combining two different tree rearrangement methods was used: nearest neighbor interchange (NNI) and subtree pruning and regrafting (SPR). The reliability of the obtained topology was estimated with the approximate likelihood-ratio test (aLRT) [24]. Recombination analysis was performed by bootscanning using SimPlot [25].

For transmission network analysis, protease (PR) and reverse transcriptase (RT) sequences were concatenated in SeaView [24]. Sequences were aligned with ClustalX [21] and manually edited in MEGA [26]. Codons associated to drug resistance were stripped from the alignment to exclude convergent evolution [9]. Phylogenetic analysis was performed using a single alignment with all subtypes and CRFs included as previously described [19], [27]. Best-fit model was chosen with Modeltest v3.7 under the Akaike information criterion [23]. ML tree was constructed in PhyML incorporated in Bioportal server [28]. Reliability of the tree was assessed using bootstrap replication (1000 replicates). Genetic distance for clusters with bootstrap support ≥90% was measured in MEGA [26]. Sequences with genetic distance <0.05 (range 0.000–0.049) substitutions per site were considered genetically related and patients were assumed to belong to the same transmission cluster [29]. Automatic cluster detection was used to confirm the transmission clusters that were initially detected. This was performed with PhyloPart program based on Approximate ML tree obtained with FastTree program [30]. Clusters were detected through the depth-first search of reliable nodes with patristic distance under 1^st^ percentile threshold of the whole tree distance [31]. According to this threshold, transmission clusters were recognized with patristic distance <0.07 substitutions per site and node reliability ≥90%.

### Resistance mutation analysis

Resistance mutation analysis was performed using the Stanford genotypic resistance interpretation algorithm [32]. Mutations specifically associated with transmitted HIV-1 drug resistance were analyzed with the Calibrated Population Resistance Tool (CPR) (http://cpr.stanford.edu) [33], [34].

### Statistical analysis

Statistical analysis was performed with GraphPad Prism version 5.00 for Windows, (GraphPad Software). The Spearman rank test and linear regression analysis were used to quantify the magnitude and direction of the correlation between viral load and CD4^+^ T cells. The Mann-Whitney U test was used to compare independent groups. The frequencies of drug resistance mutations of Angolan viruses were compared with those available at the Stanford HIV Drug Resistance Database [32] for the same subtypes using Fisher's exact test. *P*-values <0.05 were considered significant.

### GenBank accession numbers

Sequences have been assigned the following GenBank accession numbers: KF853612–KF853892.

## Results

### HIV-1 genetic diversity

Plasma samples were obtained from 139 HIV-1 infected individuals. The mean age of the patients was 36 years (SD, 14) and most (62.6%) were women (Table 1). The main route of transmission was heterosexual contact (86.3%). As expected, plasma viral load was high in most patients (mean 5.1 log_10_ copies/ml) and the number of CD4^+^ T cells was low (mean, 240.5 cells/µl). Viral load and CD4^+^ T cells were negatively correlated (n = 73, Spearman r = −0.3319, P = 0.0041).

**Figure 1:**
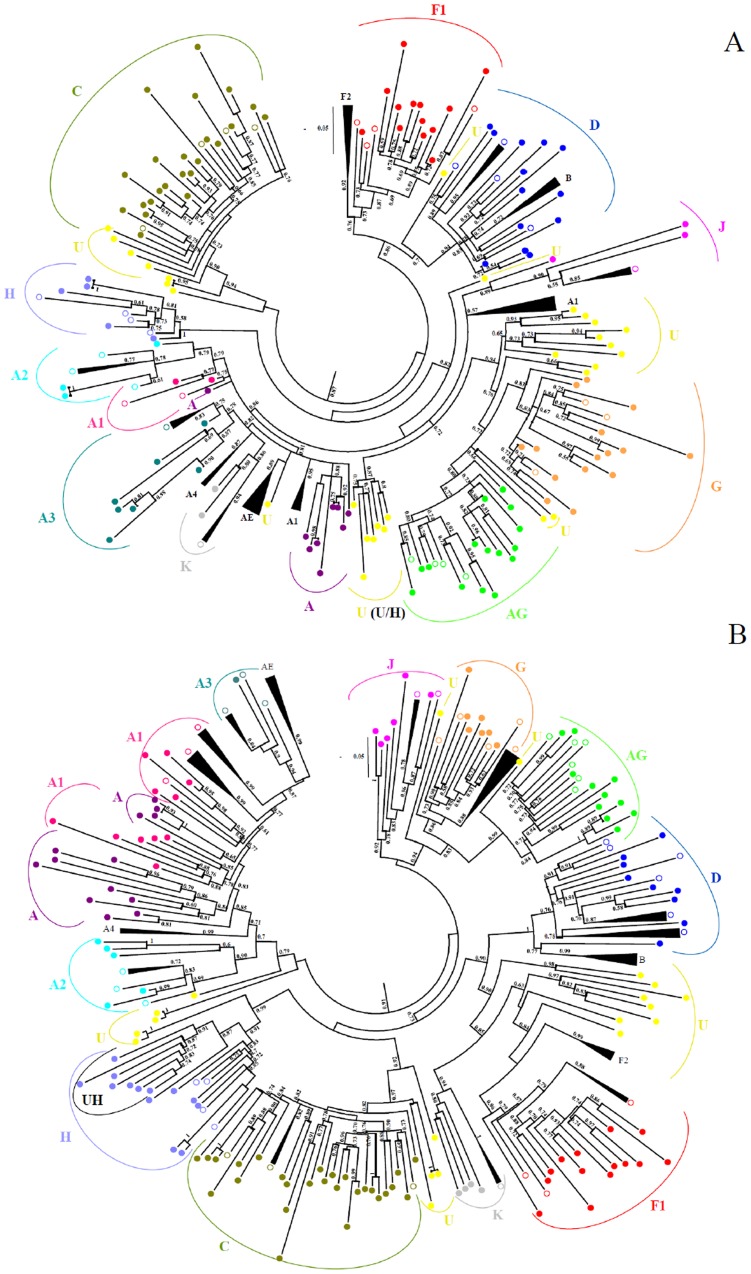
Genetic subtypes and evolutionary relationships of the viruses sequenced in this study. Maximum likelihood phylogenetic trees of PR (A) and RT (B) regions were constructed with reference sequences from all HIV-1 subtypes and sub-subtypes (empty circles) and with the Angolan sequences (filled circles). In each tree, the aLRT values supporting the internal branches defining a subtype or a sub-subtype are shown. The scale represents number of base substitutions per site.

**Figure 2:**
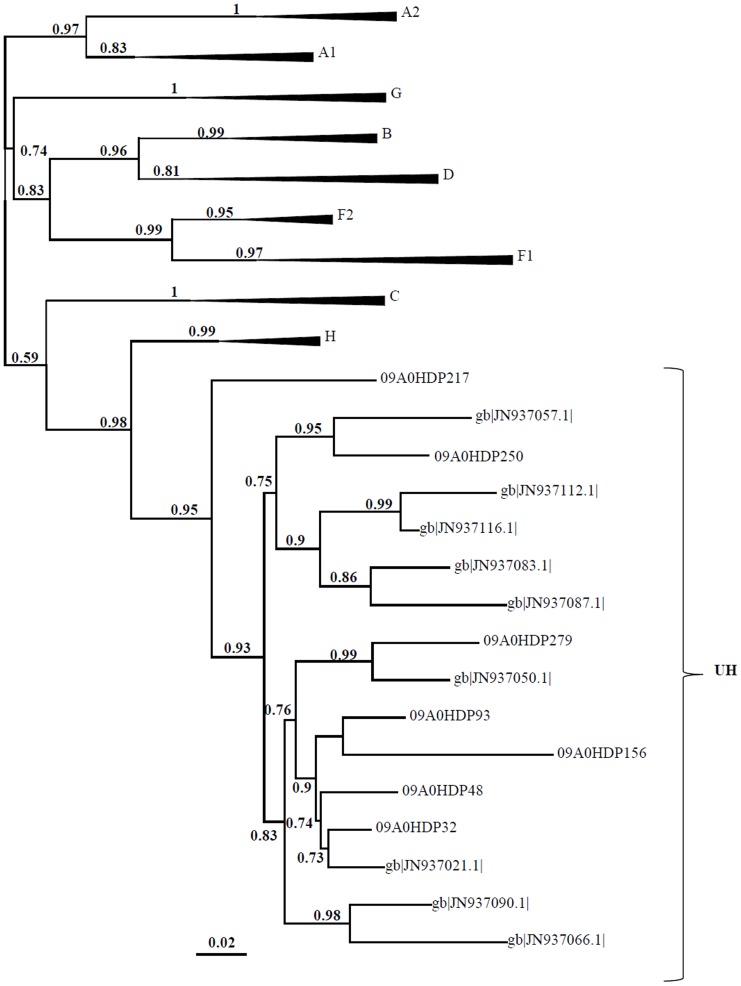
Evolutionary relationships of the U/H recombinants. Sequences of U/H recombinants (named with 09AOHDP) were aligned with those of a previous study (named gb, GenBank)[4]._ENREF_4 The aLRT values supporting the internal branches defining a subtype or a sub-subtype are shown. The scale represents number of base substitutions per site.

Sequencing and phylogenetic analysis of the PR region was completed successfully for 139 (100%) patients; RT sequences were also obtained for all but one of these patients (n = 138, 99.3%). Phylogenetic analysis showed that all viruses belonged to HIV-1 group M (Figure 1 A and B). Out of the 138 isolates for which there was PR and RT sequences, 65 (47.1%) sequences were non-recombinant and 65 (47.1%) were recombinant, of which 11 (16.9%) were CRF02_AG, and 8 (5.8%) were untypable (U). The following pure subtypes and sub-subtypes were identified: A (n = 3, 4.6%), A1 (n = 1, 1.5%), A2 (n = 3, 4.6%), C (n = 24, 36.9%), D (n = 9, 13.8%), F1 (n = 13, 20.0%), G (n = 7, 10.8%), H (n = 3, 4.6%) and J (n = 2, 3.2%). Thirty different patterns of recombination were found. Subtypes A1, A2, A3, C, D, F1, G, H, J and K, and CRF02_AG and U sequences were involved in recombination events. Most of the recombinants (n = 54, 83.1%) were URFs; in almost half of the recombinants (n = 31, 47.7%) one of the regions was untypable. The most frequent URFs comprised untypable sequences with subtypes H (U/H, n = 7, 10.8%), A (U/A, n = 6, 9.2%) and G (G/U, n = 4, 6.2%). The U/H recombinants had a mean genetic distance of 0.062 substitutions per site and formed a highly supported monophyletic cluster in both genomic regions indicating that they share the same origin (Figure 1A and B). A similar U/H cluster has been described recently in Angola but the Province of origin of the patients in this cluster has not been disclosed [4]. Phylogenetic analyses revealed a close evolutionary relatedness of all U/H sequences suggesting that the origin of this emerging URF is Luanda (Figure 2).

The analyses of the evolution of HIV-1 genetic diversity in Luanda from 2001 to 2009, showed that there was a significant decrease in the prevalence of subtype A (3.7 fold difference, P = 0.0019) which was replaced by subtype C as the dominant subtype (Table 2). Moreover, the percentage of URFs increased more than twice during the same period (P<0.0001).

**Table 2:** Evolution of HIV-1 genetic diversity in Luanda from 2001 to 2009.

HIV-1 genetic forms	2001	2009	P value^a^
	n (%)	n (%)	
**Pure subtypes**	46/86 (53.5)	65/138 (47.1)	0.6771
**Recombinants**	40/86 (46.5)	65/138 (47.1)	
**First generation CRFs**	5/40 (16)	11/65 (16.9)	0.5902
**URFs**	16/40 (40)	54/65 (83.1)	**<0.0001**
**Prevalence of Subtype A**	17/46 (40)	7/65 (10.8)	**0.0019**
**Prevalence of Subtype C**	15/46 (33)	24/65 (36.9)	0.6899
**Prevalence of Subtype F**	6/46 (13)	13/65 (20)	0.4453

a - Fisher's exact test.

### Drug resistance mutations and other polymorphisms

There were no major mutations associated with resistance to protease inhibitors (PIs). The minor resistance mutations L10I and L10V, associated with resistance to most of the PIs when present with other mutations [35], [36], were found in 15.7% and 17.6% of the isolates, respectively (Table S1). This is higher than the frequencies previously described for untreated patients (6.8% and 8.2%) [32]. V11I, associated with resistance to darunavir [36], [37], was detected in 7.7% of subtype F isolates and 13.3% of CRF02_AG isolates. This frequency is significantly higher than that found in sequences of the same subtypes available in the Stanford Database [32] (Table S1). K20I was found in almost all G and CRF02_AG isolates and is a natural polymorphism of both genetic forms [38]. K20V was found in one patient harboring a CRF02_AG virus. K20I/V codons are nonpolymorphic in most subtypes [32], [39]. They appear to be selected most commonly by nelfinavir and to reduce its susceptibility [40], [41]. A71T was found in one patient infected with a subtype C virus and A71V was found in two patients infect with subtype D. The latter mutation has never been described for subtype D [32]. A71T/V are polymorphisms that occur in 2–3% of untreated individuals but the frequency increases in patients receiving PIs [42], [43], [44]. In subtype D isolates the frequency of polymorphism I13V was significantly lower than that found in sequences of the same subtype available in the Stanford database [32] (Table S1). Similar findings were obtained for K14R, E35D and R57K in subtype A and for L89M in subtype G. For all the other polymorphisms the frequencies found in the Angolan isolates were significantly higher when compared with the worldwide sequences available from untreated patients [32]. Subtype F isolates were the most polymorphic followed by subtype A and CRF02_AG.

In the RT, we detected the K103N mutation in one patient (1/138, 0.7%) that was infected with a subtype G virus (Table S2). This mutation confers high-level resistance to nevirapine, delavirdine and efavirenz [32]. In subtype F the frequency of polymorphisms A272P and I326V was significantly lower than that found in sequences of the same subtype available in the Stanford Database [32]. Similar findings were obtained for K11T, D123AS, K173S, Q174K, V179I, Q207E, R211S, T286A, E312D and G335DE in subtype A, I293V, I329L and G335D in subtype C and T200A, V292I and G335D in CRF02_AG. For all the other polymorphisms the frequencies found in the Angolan isolates were significantly higher when compared with the sequences available from untreated patients worldwide. Compared with the 2001 survey there was a 2.3-fold decrease in the prevalence of TDR (1.6% vs 0.7%).

### Transmission network analysis

To better characterize the dynamics of the current HIV-1 epidemics in Angola and assist in the implementation of more focused prevention strategies we performed a transmission network analysis. The majority of the 364 sequences included in this sub-study were from patients residing in Luanda (n = 202, 55.5%); the remaining sequences were derived from patients from seven other provinces of Angola (n = 46, 12.6%) or their origin was unknown (n = 116, 31.9%).

**Figure 3:**
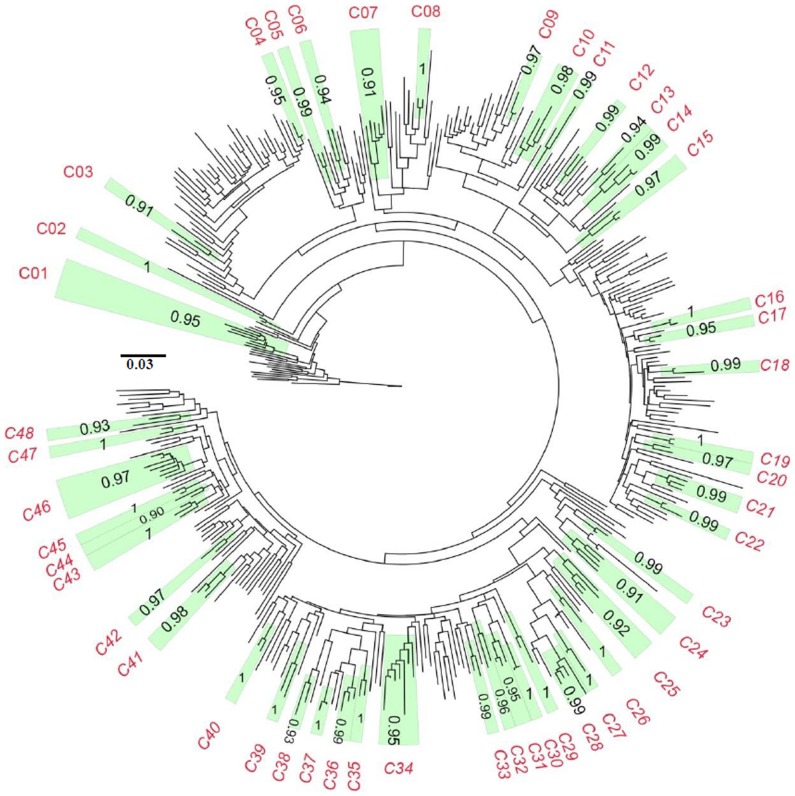
Transmission cluster analysis. Maximum likelihood tree with the 48 transmission clusters colored in green. Maximum likelihood tree was constructed in PhyML. Reliability of the tree was assessed using bootstrap resampling (1000 replicates). Bootstrap values and cluster number are indicated in each cluster. A bootstrap value of 0.7 (70%) or greater indicate significant support for the clusters. The scale represents number of base substitutions per site.

Forty eight transmission clusters were identified comprising 130 patient sequences (35.7% of the sampled patients) (Figure 3); more than half of these (52.3%) reported being heterosexual (Table S3). Consistent with this, small clusters comprising two closely related strains were dominant (n = 33, 68%). Only three large transmission chains, each comprising seven individuals, were found.

As expected, most sequences (n = 98, 75.4%) in the transmission clusters were sampled in 2008, 2009 and 2010 and most clusters (N = 27, 56.3%) comprised only sequences sampled in these dates (Table S3). All but one sequence (from Namibe) with available information on the origin were from Luanda indicating that the current epidemic is mostly sustained by local transmission. Notably, 14 (29.2%) transmission clusters comprised 2001 and 2008–2010 sequences suggesting a long standing presence of some of the more current viruses circulating in Luanda. In 3 (21.4%) of these clusters 2001 sequences originated from Cabinda, Benguela and Lunda Norte Provinces.

Six clusters (12.5%) comprised only sequences sampled in 2001 and one cluster (2.1%) comprised only sequences sampled in Cabinda in 1993 (Table S3). These clusters were considered uninformative for the current study.

Finally, in 27 transmission pairs sampled in the same year (including pairwise clusters within larger transmission chains) very low genetic distance was found (median 0.005 nucleotide substitutions per site; range 0.000–0.007) suggesting recent transmission events [45]. Potential sample mix-up for pairwise clusters with 0.000 genetic distances was excluded based on visual inspection of pairwise alignments and origin of the samples. The main features of the individuals included in the transmission networks were no different compared to individuals outside the transmission networks (Table 3).

**Table 3:** Demographic, immunologic and virologic characteristics of HIV-1 patients included in the transmission networks and patients outside of the networks.

Variables		Total	Transmission networks	Remaining patients	P value
Patients [n (%)]		364 (100)	130 (35.71)	234 (64.28)	-
Gender [n (%)]					
	Male	100 (27.47)	44 (33.85)	56 (23.93)	
	Female	142 (39)	47 (36.15)	95 (40.6)	0.1250^a^
	Unknown	122 (33.52)	39 (30)	83 (35.47)	
Transmission route [n (%)]					
	Heterosexual	194 (53.3)	68 (52.3)	126 (53.85)	
	Vertical	15 (4.12)	7 (5.38)	8 (3.42)	0.8208^a^
	Others	10 (2.74)	4 (3.08)	6 (2.56)	
	Unknown	145 (39.83)	51 (39.23)	94 (40.17)	
Origin [n (%)]					
	Luanda	200 (54.94)	78 (60)	122 (52.14)	
	Cabinda	20 (5.49)	4 (3.08)	16 (6.84)	0.2850^a^
	Others	28 (7.69)	8 (6.15)	20 (8.55)	
	Unknown	116 (31.87)	40 (30.77)	76 (32.48)	
CD4 [mean (range), cells/µl]			335.39 (1-1914) (n = 41)	300.12 (12–790) (n = 78)	0.7309^b^
HIV RNA [mean, (range) log_10_, copies/ml]			5.15 (1.94–7) (n = 33)	5.06 (1.6–6.68) (n = 52)	0.6391^b^

a – Chi-square test; b – Mann-Whitney test;

## Discussion

We assessed HIV-1 diversity, transmission dynamics and prevalence of TDR in Luanda in 2009, five years after the scale-up of ART and compared these data with our previous survey performed in 2001 [6], [15]. Individuals included in this study had a low CD4 count which was directly related with high viral load. These features are consistent with the reported absence of ART [46], [47].

Like in 2001, no major PIs resistance mutations were found in the study population which is consistent with the fact that first-line regimens used in Angola do not include PIs [48]. However, some minor mutations in the PR and many unusual polymorphisms were detected suggesting that some Angolan isolates might have a low genetic barrier for resistance to some PIs [32]. The K103N mutation, which confers high-level resistance to nevirapine, delavirdine and efavirenz [32], was found in one patient accounting for a 0.7% prevalence rate of TDR which is 2.3 fold lower compared to the 2001 survey [15]. This residual TDR prevalence is similar to that of several African countries that also use the public health approach to ART [49], [50], [51], [52], [53] and suggests that the most common first-line ARV regimens will be effective in this population.

Similar to previous studies, the HIV-1 epidemic in Luanda in 2009 was highly complex being characterized by the presence of almost all subtypes (A, C, D, F, G, H and J; 47.1%), complex recombinants viruses (47.1%) and untypable (5.8%) strains [3], [4], [5], [6], [54]. A high number of our sequences fall at basal positions on the phylogenetic trees (pre-subtype branches) which is consistent with the long standing presence of HIV-1 in Angola [6]. In addition, some strains from Angola have little organized substructure and form weaker clusters within phylogenetic trees than the global reference sequences, not allowing a clear distinction between subtypes. As a consequence, the current global subtype classification may not reflect the extent of diversity in this region [55].

The prevailing subtype in 2009 in Luanda was subtype C (36.9%) followed by sub-subtype F1 (20.0%) whereas in 2001 it was subtype A followed by subtype C [6]. The significant decrease in the prevalence of subtype A and increase in subtype C observed in 2009 could be explained by the increasing predominance of subtype C in the bordering countries, namely in the south region of Democratic Republic of Congo [56] and in Zambia [51], [57], [58]. As in 2001, almost half of our sequences were recombinant comprising all group M subtypes as well as CRF02_AG and U sequences. The frequency of CRFs did not change between 2001 and 2009, but the frequency of URFs more than doubled in the same period. This is on contrast to the global and regional distribution of HIV-1 genetic forms between 2000 and 2007, where there was a notable increase in the proportion of CRFs and a decrease in URFs [59]. Importantly, the results indicate that the Angolan HIV-1 epidemic is still increasing in genetic complexity and suggest high rates of co-infection and/or superinfection [60] which is consistent with an increasing HIV-1 incidence and prevalence [1], [2], [61]. The most common URF was U/H found in seven strains (10.8% of the recombinants and 5.1% of the total population). This new recombinant strain was found in unrelated patients and its sequences clustered in a highly supported monophyletic group suggesting that it was originally produced in Luanda. The close relationship with U/H sequences recently reported elsewhere in Angola [4], indicates that this new recombinant is already established in Angola. Sequencing the full-length genome of this recombinant strain will be needed to determine if this is a new CRF.

A large number of transmission clusters were identified in this study which included 35.7% of the analyzed samples. This is not uncommon in HIV epidemics as within a smaller population or even globally HIV infected individuals are often part of wide transmission networks [16], [19], [62]. Small clusters mostly comprising two sequences were dominant over large clusters which is consistent with heterosexual contact being the main route of transmission reported in most patients [63]. While most transmission clusters comprised only sequences from Luanda and were therefore consistent with a locally propelled epidemic, some clusters contained sequences from Luanda and from other locations in Angola consistent with a more complex origin and transmission dynamics going well beyond the borders of the capital city. Finally, based on high sequence homology between patients in transmission clusters, a large number of potential recent infections were inferred. Overall, the results are consistent with a rising HIV-1 epidemic in Luanda [1], [2], [61]. Further surveys are required to obtain a clearer picture of the dynamics of the current HIV-1 epidemics at the national level.

In conclusion, transmission of drug resistant strains was still negligible in Luanda in 2009, five years after the scale-up of ART. The dominance of small and recent transmission clusters and the emergence of new URFs are consistent with a rising HIV-1 epidemics mainly driven by heterosexual transmission.

## Supporting Information

Table S1 **Minor mutations and natural polymorphisms detected in the PR of drug-naive patients from Luanda.**
(XLS)

Table S2 **Drug resistance mutations and natural polymorphisms detected in the RT of drug-naive patients from Luanda.**
(XLS)

Table S3 **Epidemiological characteristics of the patients included in the transmission clusters.**
(DOC)
