# Supplementary material for: HIV-1 Diversity, Transmission Dynamics and Primary Drug Resistance in Angola
Source: PLoS One. 2014 Dec 5;9(12):e113626. doi: 10.1371/journal.pone.0113626 (PMC4257534; doi:10.1371/journal.pone.0113626)
Supplement: Table S3 — Epidemiological characteristics of the patients included in the transmission clusters. (DOC) [file pone.0113626.s003.doc]

**Table S3**- Epidemiological characteristics of the patients included in the transmission clusters

| **Cluster number** | **Sequence** | **Sampling date** | **Age** | **Gender** | **Reported transmission route** | **Region** |
| --- | --- | --- | --- | --- | --- | --- |
| 1 | 09AGHDP119 | 2009 | 1980 | F | Heterosexual | Luanda |
|  | 09AGHDP226 | 2009 | n.a. | F | n.a. | Luanda |
|  | 09AGHDP208 | 2009 | 1970 | F | Heterosexual | Luanda |
|  | JN937038 | 2008 | 1975 | F | n.a. | n.a. |
|  | 01AOHM176 | 2001 | 1963 | M | Heterosexual | Luanda |
|  | JQ616884 | 2009 | n.a. | n.a. | n.a. | n.a. |
|  | JN937034 | 2008 | n.a. | n.a. | n.a. | n.a. |
| 2 | JQ616880 | 2009 | n.a. | n.a. | n.a. | n.a. |
|  | JN937047 | 2009 | n.a. | n.a. | n.a. | n.a. |
| 3 | 09AGHDP237 | 2009 | 1957 | M | Heterosexual | Luanda |
|  | 01AOCSE126 | 2001 | 1976 | F | n.a. | Lunda Norte |
| 4 | 01AOLFA13 | 2001 | 1971 | M | Heterosexual | Luanda |
|  | 01AOHAB86 | 2001 | 1963 | F | n.a. | Luanda |
| 5 | JN937098 | 2010 | n.a. | n.a. | n.a. | n.a. |
|  | JN937104 | 2010 | n.a. | n.a. | n.a. | n.a. |
| 6 | 09AGHDP186 | 2009 | 1982 | F | Heterosexual | Luanda |
|  | JQ616882 | 2009 | n.a. | n.a. | n.a. | n.a. |
| 7 | 09AGHDP62 | 2009 | 1973 | F | Heterosexual | Luanda |
|  | JN937097 | 2010 | n.a. | n.a. | n.a. | n.a. |
|  | 01AOSNS09 | 2001 | 1981 | F | Homosexual | Luanda |
|  | 09AGHDP42 | 2009 | 1967 | M | Heterosexual | Luanda |
|  | 09AGHDP289 | 2009 | 1985 | F | Heterosexual | Luanda |
| 8 | 93AOHDC247 | 1993 | n.a. | F | n.a. | Cabinda |
|  | 93AOHDC253 | 1993 | n.a. | F | n.a. | Cabinda |
| 9 | JN937116 | 2010 | n.a. | n.a. | n.a. | n.a. |
|  | JN937112 | 2010 | 1976 | M | Heterosexual | Central |
| 10 | JN937050 | 2009 | n.a. | n.a. | n.a. | n.a. |
|  | JQ616883 | 2009 | n.a. | n.a. | n.a. | n.a. |
|  | 09AGHDP279 | 2009 | 1960 | M | Heterosexual | Luanda |
|  | 01AOHJM06 | 2001 | 1968 | F | Heterosexual | Luanda |
| 11 | 01AOSNS01 | 2001 | 1959 | M | Heterosexual | Luanda |
|  | 01AOHDP73 | 2001 | 1958 | M | Heterosexual | Luanda |
| 12 | JN937046 | 2009 | n.a. | n.a. | n.a. | n.a. |
|  | JN937037 | 2008 | n.a. | n.a. | n.a. | n.a. |
| 13 | JN937061 | 2009 | n.a. | n.a. | n.a. | n.a. |
|  | JN937101 | 2010 | n.a. | n.a. | n.a. | n.a. |
| 14 | 09AGHDP233 | 2009 | 1979 | M | Heterosexual | Luanda |
|  | 09AGHDP280 | 2009 | 2004 | M | Vertical | Luanda |
|  | JN937054 | 2009 | 1964 | F | Heterosexual | Central |
|  | 01AOSNS56 | 2001 | 1965 | M | n.a. | Luanda |
| 15 | 09AGHDP231 | 2009 | 1987 | F | Heterosexual | Luanda |
|  | 09AGHDP164 | 2009 | 1992 | F | Heterosexual | Luanda |
|  | 01AOHDC229 | 2001 | 1964 | M | Heterosexual | Cabinda |
| 16 | 09AGHDP44 | 2009 | 2007 | M | Vertical | Luanda |
|  | 09AGHDP68 | 2009 | 1985 | F | Heterosexual | Luanda |
| 17 | 09AGHDP50 | 2009 | 1963 | M | Heterosexual | Luanda |
|  | 09AGHDP118 | 2009 | 1978 | F | Heterosexual | Luanda |
| 18 | 09AGHDP242 | 2009 | 1970 | M | Heterosexual | Luanda |
|  | 09AGHDP111 | 2009 | 1965 | F | Heterosexual | Luanda |
| 19 | JN937017 | 2008 | 1987 | F | Heterosexual | Namibe |
|  | JN937040 | 2008 | 1967 | M | MSM | Luanda |
| 20 | JN937095 | 2010 | 1965 | M | Heterosexual | Benguela |
|  | 01AOHDP71 | 2001 | 1949 | F | Heterosexual | Luanda |
| 21 | 01AOHJM64 | 2001 | 1975 | F | n.a. | Luanda |
|  | 01AOSNS55 | 2001 | 1966 | F | n.a. | Luanda |
|  | 09AGHDP240 | 2009 | 1982 | F | Heterosexual | Luanda |
| 22 | 09AGHDP74 | 2009 | 1939 | M | Heterosexual | Luanda |
|  | JQ616899 | 2009 | n.a. | n.a. | n.a. | n.a. |
| 23 | 09AGHDP94 | 2009 | 1973 | M | Heterosexual | Luanda |
|  | 09AGHDP30 | 2009 | 2007 | M | Vertical | Luanda |
| 24 | JN937059 | 2009 | n.a. | n.a. | n.a. | n.a. |
|  | JN937060 | 2009 | n.a. | n.a. | n.a. | n.a. |
|  | JN937115 | 2010 | 1981 | F | n.a. | Central |
|  | 09AGHDP82 | 2009 | 2006 | M | Vertical | Luanda |
| 25 | 09AGHDP212 | 2009 | 1981 | F | Heterosexual | Luanda |
|  | 01AOSNS04 | 2001 | 1969 | M | Bisexual | Luanda |
|  | JQ616913 | 2009 | n.a. | n.a. | n.a. | n.a. |
|  | 09AGHDP263 | 2009 | 1978 | F | Heterosexual | Luanda |
| 26 | 09AGHDP267 | 2009 | 1971 | M | Heterosexual | Luanda |
|  | 01AOSNS24 | 2001 | 1973 | F | Heterosexual | Luanda |
| 27 | 01AOLFA90 | 2001 | 1977 | M | n.a. | Luanda |
|  | 01AOSNS49 | 2001 | 1957 | M | Bisexual | Cabinda |
| 28 | JN937081 | 2010 | n.a. | n.a. | n.a. | n.a. |
|  | JN937070 | 2010 | n.a. | n.a. | n.a. | n.a. |
|  | JN937073 | 2010 | n.a. | n.a. | n.a. | n.a. |
|  | JN937079 | 2010 | n.a. | n.a. | n.a. | n.a. |
| 29 | 09AGHDP169 | 2009 | 1986 | M | Heterosexual | Luanda |
|  | 09AGHDP167 | 2009 | 1980 | M | Heterosexual | Luanda |
| 30 | 09AGHDP130 | 2009 | 1984 | F | Heterosexual | Luanda |
|  | 09AGHDP145 | 2009 | 1976 | M | Heterosexual | Luanda |
| 31 | 01AOLFA19 | 2001 | 1963 | M | Heterosexual | Luanda |
|  | 01AOLFA17 | 2001 | n.a. | F | Heterosexual | Luanda |
|  | JQ616890 | 2009 | n.a. | n.a. | n.a. | n.a. |
| 32 | 09AGHDP49 | 2009 | 1973 | M | Heterosexual | Luanda |
|  | 01AOCSE136 | 2001 | 1972 | M | Heterosexual | Luanda |
| 33 | JQ616894 | 2009 | n.a. | n.a. | n.a. | n.a. |
|  | JQ616891 | 2009 | n.a. | n.a. | n.a. | n.a. |
| 34 | 09AGHDP64 | 2009 | 1963 | F | Heterosexual | Luanda |
|  | 09AGHDP200 | 2009 | 1969 | M | Heterosexual | Luanda |
|  | JN937085 | 2010 | n.a. | n.a. | n.a. | n.a. |
|  | 09AGHDP296 | 2009 | 1976 | F | Heterosexual | n.a. |
|  | 01AOLFA94 | 2001 | 1969 | M | Heterosexual | Luanda |
|  | 01AOSNS36 | 2001 | n.a. | F | Heterosexual | Luanda |
|  | JN937075 | 2010 | n.a. | n.a. | n.a. | n.a. |
| 35 | 09AGHDP281 | 2009 | 1978 | F | Heterosexual | Luanda |
|  | JQ616886 | 2009 | n.a. | n.a. | n.a. | n.a. |
| 36 | 01AOSNS03 | 2001 | 1964 | M | Heterosexual | Luanda |
|  | 01AOLFA14 | 2001 | 1956 | M | Heterosexual | Luanda |
|  | 01AOLFA18 | 2001 | 1958 | F | Heterosexual | Luanda |
| 37 | 09AGHDP274 | 2009 | 1976 | M | Heterosexual | Luanda |
|  | 09AGHDP204 (a2) | 2009 | 2006 | F | Vertical | Luanda |
| 38 | 09AGHDP106 | 2009 | 2006 | M | Vertical | Luanda |
|  | 09AGHDP57 | 2009 | 1966 | F | Heterosexual | Luanda |
| 39 | 09AGHDP290 | 2009 | 1980 | M | Heterosexual | Luanda |
|  | JN937058 | 2009 | n.a. | n.a. | n.a. | n.a. |
| 40 | 09AGHDP100 | 2009 | 1969 | F | Heterosexual | Luanda |
|  | JN937033 | 2008 | n.a. | n.a. | n.a. | n.a. |
| 41 | JN937035 | 2008 | n.a. | n.a. | n.a. | n.a. |
|  | JN937072 | 2010 | n.a. | n.a. | n.a. | n.a. |
|  | 09AGHDP37 | 2009 | 1972 | F | Heterosexual | Luanda |
| 42 | 09AGHDP157 | 2009 | 1958 | M | Heterosexual | Luanda |
|  | JQ616905 | 2009 | n.a. | n.a. | n.a. | n.a. |
| 43 | JQ616885 | 2009 | n.a. | n.a. | n.a. | n.a. |
|  | 09AGHDP20 | 2009 | 1982 | F | Heterosexual | Luanda |
|  | 09AGHDP245 | 2009 | 1989 | F | Heterosexual | Luanda |
| 44 | 01AOCSE125 | 2001 | 1969 | M | Heterosexual | Luanda |
|  | 01AOHDP75 | 2001 | 1973 | F | Heterosexual | Luanda |
| 45 | JN937031 | 2008 | n.a. | n.a. | n.a. | n.a. |
|  | JN937106 | 2010 | 1991 | F | Heterosexual | Central |
| 46 | 09AGHDP258 | 2009 | 2006 | M | Heterosexual | Luanda |
|  | 09AGHDP266 | 2009 | 1986 | F | Heterosexual | Luanda |
|  | 01AOSNS40 | 2001 | 1976 | F | Heterosexual | Luanda |
|  | 09AGHDP86 | 2009 | 2006 | M | Vertical | Luanda |
|  | JN937028 | 2008 | n.a. | n.a. | n.a. | Central |
|  | JQ616898 | 2009 | n.a. | n.a. | n.a. | n.a. |
|  | 09AGHDP105 | 2009 | 1976 | M | Heterosexual | Luanda |
| 47 | 09AGHDP201 | 2009 | 1951 | M | Heterosexual | Luanda |
|  | JQ616897 | 2009 | n.a. | n.a. | n.a. | n.a. |
| 48 | 01AOLFA97 | 2001 | 1958 | F | Heterosexual | Luanda |
|  | 01AOSNS37 | 2001 | 1973 | F | n.a. | Luanda |

n.a.- not available
